# Modified heptapeptide from tau binds both tubulin and microtubules

**DOI:** 10.1111/1759-7714.13643

**Published:** 2020-09-07

**Authors:** Jingrui Li, Yuyang Li, Min Liu, Songbo Xie

**Affiliations:** ^1^ College of Life Sciences, Shandong Provincial Key Laboratory of Animal Resistance Biology, Collaborative Innovation Center of Cell Biology in Universities of Shandong, Institute of Biomedical Sciences Shandong Normal University Jinan China

**Keywords:** Affinity, cell‐penetrating peptide, microtubule, tau‐derived peptide, tubulin

## Abstract

**Background:**

Microtubules are the major cytoskeletal component in eukaryotes which are essential for a large spectrum of cellular activities. Monitoring the behavior of microtubules is helpful for a better understanding of the regulatory mechanism governing microtubule architecture and microtubule‐based activities. Here, we characterized the binding capability of a modified heptapeptide from tau to both tubulin and microtubules and sought to develop it as a fluorescent peptide for monitoring microtubules.

**Methods:**

To deliver the fluorescent peptide into the cells, a cell‐penetrating peptide was conjugated to the modified heptapeptide from tau and synthesized. The affinity of the modified heptapeptide was determined by microscale thermophoresis. The microtubule labeling ability was determined by adding the peptide into the polymerized microtubule solutions or cultured HeLa cells.;

**Results:**

Affinity determination revealed that the tau‐derived peptide specifically bound to tubulin. In addition, the peptide was able to label polymerized microtubules in solution, although no obvious microtubule filaments were observed clearly in living cells, probably due to the inadequate affinity.

**Conclusions:**

These results suggest that using a peptide‐based strategy for imaging microtubules might be plausible and attempts to improve its affinity is warranted in the future.

## Graphical abstract

We characterized the binding capability of a seven‐amino‐acid peptide derived from tau to microtubules and sought to develop it as a fluorescent peptide for monitoring microtubules.

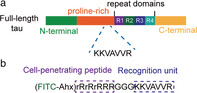

## Introduction

Microtubules, composed of α‐ and β‐tubulin heterodimers, are the major cytoskeletal component in eukaryotes.^1^ Alone or together with actin and intermediate filaments, they participate in a diversity of cellular activities, including cell shape maintenance, cell motility, mitotic division, cell junction, and cell differentiation.^2^, ^3^, ^4^, ^5^, ^6^ To fulfill these activities, microtubules reorganize their architecture dynamically to adapt to the cellular requirement. Although the principal mechanism governing microtubule‐based architecture and activity has been dissected through fusing fluorescence protein tags into tubulin or end‐binding (EB) proteins, such fusion proteins suffer from intrinsic limitations.^7^ For example, the transfection efficiency is extremely low in primary cells; the physiological microtubule function is perturbed by the large size of fluorescence protein tags. Therefore, development of a straightforward, small size molecule to trace microtubule behavior is necessary to better understand the subtle behaviors of microtubules and their regulatory mechanism.

The property of microtubules is regulated by a number of microtubule‐binding proteins,^8^, ^9^, ^10^, ^11^, ^12^, ^13^ of which tau is well‐characterized because of its abundance, particularly in brain tissues.^14^, ^15^, ^16^ The N‐terminus of tau is a projection domain, followed by a proline‐rich region and a C‐terminal microtubule‐assembly domain.^17^ The microtubule‐assembly domain, which contains four imperfect sequence repeats, is responsible for the binding of tau to microtubules. Indeed, the microtubule‐assembly domain fused with GFP is able to trace microtubules in cells;^18^, ^19^ however, it has some adverse effects on microtubule dynamics due to the large size of the fusion protein. To avoid this issue, here we sought to develop a fluorescent tau‐derived peptide with small size and delivered it into cells via a cell‐penetrating peptide strategy.

## Methods

### Materials

Adenosine triphosphate, guanosine 5′‐triphosphate (GTP) sodium salt hydrate, and benzonase were purchased from Sigma‐Aldrich. Paclitaxel was purchased from Cell Signaling Technology. The tumor overexpressed gene (TOG) column was prepared as previously described.^20^ Capillary tubes were purchased from Nano Temper. GSTrap column was purchased from GE Healthcare. Tetramethylrhodamine was purchased from Thermo Fisher.

### Peptide synthesis

Tau‐derived and scrambled control peptides were synthesized with the solid‐phase methodology using the standard Fmoc chemistry by GenScript (Nanjing, China). N‐terminal fluorescein‐labeling was performed as previously described.^21^ The synthesized peptides were purified by high performance liquid chromatography using a C‐18 reversed phase column and then analyzed by mass spectrometry.

### Affinity determination

All microscale thermophoresis measurements were performed using Monolith NTTM hydrophobic capillaries and a Monolith NT.115 instrument (NanoTemper) according to the manufacturer's instructions. The peptide was prepared with varying concentrations for pretest to ensure final proper concentration. The fluorescence intensity value should be more than 200. A series of dilution of the tubulin dimers was then added into the capillaries. The affinity of the peptide to tubulin dimers was examined using the Monolith NT.115 instrument.

### Tubulin purification

Tubulin from HeLa cells was purified using a TOG‐based affinity column.^20^ Briefly, cells were resuspended in an equal volume of BRB80 buffer containing 3 μL of benzonase and 1 mM DTT. Cells were lysed on ice and centrifuged at 40 000 rpm for 30 minutes at 4°C. The supernatant was filtered through a 0.45 μm Milliex‐HV polyvinylidene fluoride membrane, and then loaded into a TOG column preequilibrated with BRB80 buffer. The tubulin was eluted with BRB80 buffer, and the crude extract was further purified through the cycle of polymerization and depolymerization as previously described.^22^

### Microtubule assembly

Rhodamine‐labeled tubulin (5%) was mixed into tubulin purified from HeLa cells in the PEMG buffer (100 mM PIPES, 1 mM EGTA, 1 mM MgSO_4_, 1 mM GTP, pH 6.8). Next, the mixture was placed on ice for five minutes and then incubated at 37°C for 30 minutes. The polymerized reaction was terminated by adding 400 μL of PEMG buffer. The mixture was then centrifuged at 80 000 *g* for 10 minutes. The pellet was resuspended with 300 μL of the PEMG buffer supplemented with 20 μM paclitaxel and then added into a slide chamber for imaging under a fluorescence microscope.

### Living cell assay

HeLa cells were cultured in Dulbecco's modified Eagle's medium supplemented with 10% fetal bovine serum and maintained at 37°C in a humidified 5% CO_2_ environment. The peptide was added into the Hela cells, and incubated for 30 minutes. The nuclei were stained with Hoechst 33258 (Beyotime). Cells were imaged with a fluorescence microscope (Leica, Germany).

## Results

### Design of a tau‐derived heptapeptide for monitoring microtubules

**Figure 1:**
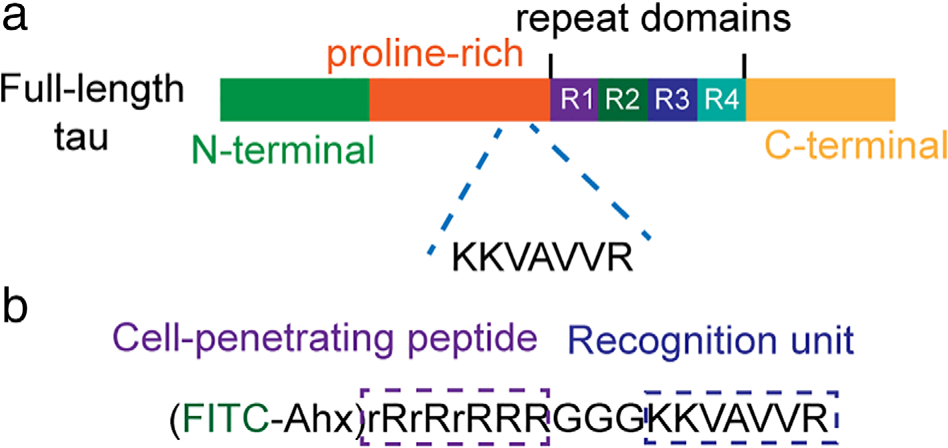
Tau‐derived peptide. (**a**) Schematic of tau domain. The peptide KKVAVVR located within the proline‐rich domain. The repeat domains contain four imperfect sequence repeats (R1–R4). (**b**) The component of fluorescent tau‐derived peptide which contains a fluorescein isothiocyanate (FITC) dye, a cell‐penetrating peptide (rR)_3_R_2_, a spacer (GGG), and a microtubule recognition unit (KKVAVVR). Ahx, e‐aminocaproic acid; r denotes D‐arginine, and R denotes L‐arginine.

Numerous studies have reported that the four repeat regions of tau are responsible for tau‐microtubule interaction.^23^, ^24^, ^25^ In addition to the repeat region, Goode *et al*. identified that a small sequence ^215^KKVAVVR^221^ within the proline‐rich region was also critical for efficient microtubule binding.^26^ In addition, Cao and Mao^27^ identified that VxxVxxP motif might be a potential microtubule‐binding region and further confirmed that the peptide KKVAVVRTPP was able to bind microtubules.^27^ Based on these findings, here we sought to develop a fluorescent peptide using this microtubule‐binding motif (Fig 1a). To deliver the peptide into cells, we added an oligoarginine (rR)_3_R_2_ (r: D‐Arg, R: L‐Arg), a widely‐used cell‐penetrating peptide,^21^ and three glycine residues as a spacer (Fig 1b). To improve the stability of fluorescein isothiocyanate (FITC), an aminohexanoic (Ahx) acid was introduced as a protective group (Fig 1b). In addition, the control peptide, which did not contain a cell‐penetrating peptide (CPP), was also synthesized.

### Modified heptapeptide binds tubulin in vitro

**Figure 2:**
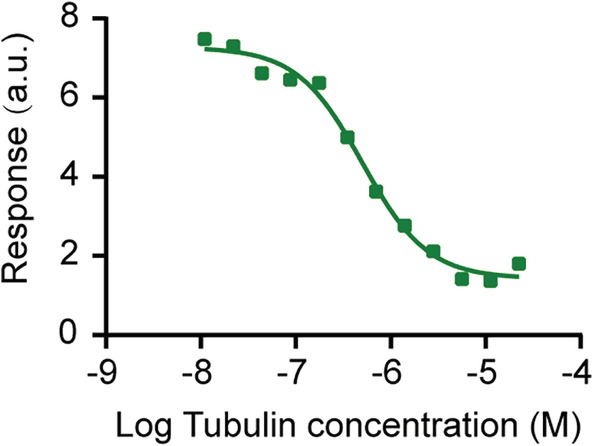
Affinity determination of the peptide to tubulin. 1 μM of the peptide and a series dilution of tubulin were added into the capillary tube, and the fluorescence intensity was then determined by a Monolith NT.115 instrument.

We then tested the affinity of the peptide with tubulin dimers by microscale thermophoresis assay.^28^, ^29^ As shown in Fig 2, the peptide bound tubulin dimers in a dose‐dependent manner, suggesting the specificity of peptide to tubulin. In addition, the disassociation constant (Kd) of the peptide with tubulin was 4.68 ± 0.08 μM, indicative of a relatively low affinity. It is noteworthy that the low affinity of the peptide to tubulin may reduce the unwanted perturbations of microtubule dynamics and physiological function, as demonstrated by Lifeact, a versatile actin marker, which shows a low affinity to F‐actin but specifically labels actin without affecting cellular processes.^30^

### Modified heptapeptide is able to label microtubules in solution but is unable to trace microtubules in living cells

**Figure 3:**
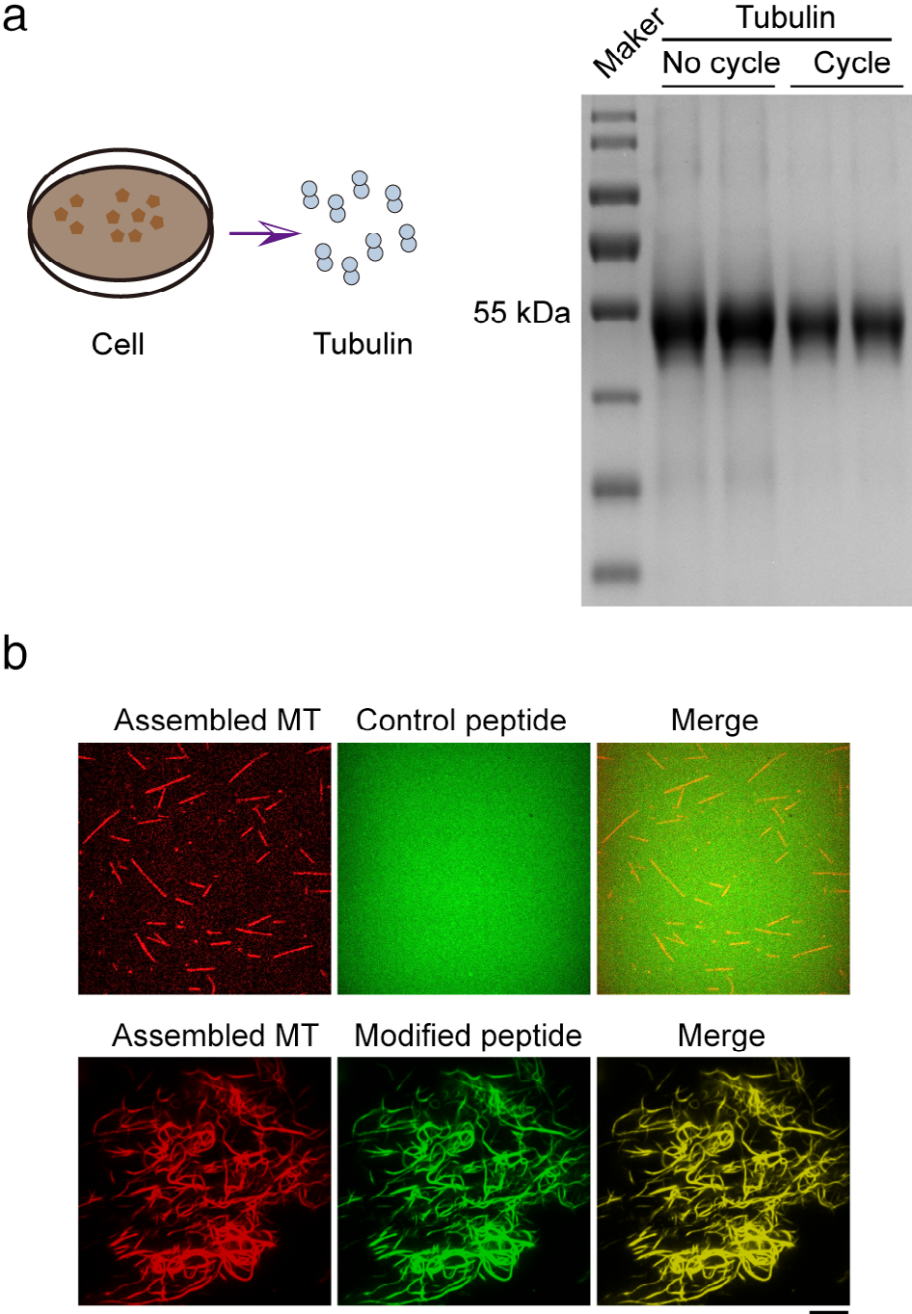
The peptide was able to label the microtubules in solution. (**a**) The tubulin was purified from HeLa cells through cycle process, and the purity was determined by SDS‐PAGE. (**b**) Rhodamine‐labeled tubulin was added into a slide chamber to first induce polymerization, and then 1 μM of the tau‐derived peptide or control peptide was added into the polymerized microtubule. After five minutes incubation, the microtubule was imaged under a fluorescence microscope. Scale bar: 5 μm.

Next, we examined whether the peptide could label polymerized microtubules in vitro. We purified assembly‐competent tubulin from HeLa cells through cycles of polymerization and depolymerization (Fig 3a). Here, we used rhodamine‐labeled tubulin for counter‐imaging of microtubules. Strikingly, the peptide was able to successfully label the microtubules in solution (Fig 3b). in vitro reconstitution of microtubule assemblies has been widely‐used for dissecting microtubule behaviors.^31^

**Figure 4:**
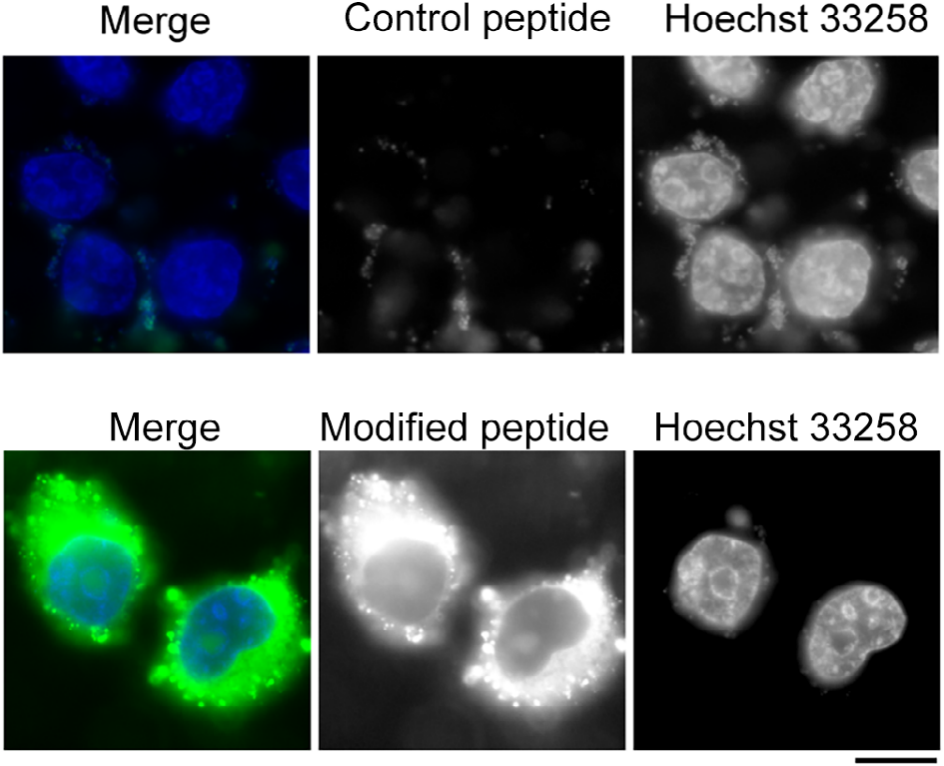
Examination of the peptide in tracing microtubules in living cells. HeLa cells were treated with 15 μM of the peptide or control peptide, together with Hoechst 33258, and 30 minutes later, the cells were imaged with a fluorescence microscope. Scale bar: 5 μm.

Last, we examined whether the peptide could efficiently enter into living cells and trace microtubules. We found that the peptide was delivered successfully into HeLa cells by cell‐penetrating peptide (rR)_3_R_2_, while the control peptide, which did not contain cell‐penetrating peptide, could not enter into cells (Fig 4). However, no clear microtubule filaments were observed, probably due to the low affinity of the peptide to microtubules. It is therefore warranted to optimize the amino residues to improve the affinity of the peptide to microtubules for living cell imaging.

## Discussion

Several reagents and methods have been developed for monitoring microtubules. 4',6‐diamidino‐2‐phenylindole (DAPI) was originally used for examining the kinetics of microtubule assembly in vitro.^32^ In addition, a fluorescent GTP analog was developed for analyzing microtubule dynamics with nanometer precision.^33^ Despite their success in monitoring microtubules in vitro, these reagents were unable to trace microtubules in living cells due to the lack of specificity and the complexity of cellular contents. Microtubule‐targeting agents, such as taxol or colchicine, were able to label microtubules in living cells due to their specificity to microtubules; however, the microtubule‐targeting agent‐based molecules are detrimental to microtubule dynamics, making it unsuitable for monitoring microtubule behaviors in living cells.^34^, ^35^, ^36^, ^37^ In this study, we sought to adopt a peptide‐based strategy to develop fluorescent molecules that monitor microtubule behavior in living cells.

Mounting evidence shows that the repeat regions are critical for the interactions of tau with microtubules. Inaba *et al*. thus developed a fluorescent peptide based on the repeat region to monitor microtubules in living cells.^38^ Although the imaging quality and delivery efficacy are struggling, it proves that the strategy of using peptides to recognize microtubule is plausible. In this study, we tested another tau‐derived peptide for its ability to monitor microtubules. Our data revealed that this modified heptapeptide from tau was able to bind both tubulins and microtubules.

In the reconstitution system, fluorophore‐labeled tubulin is often used with the intension of examination under fluorescent microscopy.^39^, ^40^ However, the conjugation of fluorophores to tubulin is tedious with great loss (the yield is no more than 10%), making it difficult to use cultured cells or mouse brain for sample preparation. The modified heptapeptide from tau binds microtubules specifically and noncovalently, providing an alternative tool for convenient microtubule labeling without the need of fluorophore conjugation, which may facilitate the investigation of microtubules from the limited source samples. Despite the unsatisfactory image of microtubules in living cells, our data indicate that using the peptide‐based strategy for imaging subcellular structures in living cell is plausible. Attempts to identify novel peptides with higher affinity is warranted in the future.

## Disclosure

No authors report any conflict of interest.
